# Correlating Various Clinical Outcomes and Associated Dispositions in Patients with Severe Traumatic Brain Injury (TBI)

**DOI:** 10.3390/life15081262

**Published:** 2025-08-08

**Authors:** Bharti Sharma, Tirth Patel, Sarah Dawson-Moroz, George Agriantonis, Munirah Hasan, Navin D. Bhatia, Carrie Garcia, Praise Nesamony, Jasmine Dave, Juan Mestre, Shalini Arora, Saad Bhatti, Zahra Shafaee, Suganda Phalakornkul, Kate Twelker, Jennifer Whittington

**Affiliations:** 1 Department of Surgery, NYC Health and Hospitals—Elmhurst, New York, NY 11373, USA; patelt12@nychhc.org (T.P.); agriantg@nychhc.org (G.A.); hassanm14@nychhc.org (M.H.); bhatian1@nychhc.org (N.D.B.); carrie.garcia@nychhc.org (C.G.); nesamonp@nychhc.org (P.N.); davej@nychhc.org (J.D.); mestreju@nychhc.org (J.M.); arorash@nychhc.org (S.A.); bhattisa@nychhc.org (S.B.); shafaeez1@nychhc.org (Z.S.); phalakos@nychhc.org (S.P.); harrisj20@nychhc.org (J.W.); 2 Department of Surgery, Icahn School of Medicine at Mount Sinai, New York, NY 10029, USA; 3 Department of Surgery, East Tennessee State University, Johnson City, TN 37614, USA; morozs@mail.etsu.edu

**Keywords:** discharge disposition, traumatic brain injury, mechanism of injury, length of stay, injury severity

## Abstract

**Background**: Traumatic brain injury (TBI) is a major cause of death and disability worldwide. Patient disposition following TBI has been shown to interact with factors such as age, sex, and injury severity to impact clinical outcomes. Discharge home is associated with better functional outcomes and lower mortality, while discharge to rehabilitation or long-term care facilities is linked to greater injury severity, older age, and higher comorbidity burden. The aim of this study was to further correlate clinical outcomes with discharge dispositions in patients with severe TBI. **Methods**: This is a retrospective study (2020–2023) of dispositions in patients with severe TBI with AIS (head) ≥ 3. We investigated the relationship between patient disposition and a range of clinical variables, using both parametric (ANOVA) and non-parametric (Kruskal–Wallis, Wilcoxon, Van der Waerden, Savage, Kolmogorov–Smirnov, and Cramer–von Mises) statistical tests. Variables significant in univariate analysis were entered into a multinomial logistic regression model, with discharge home as the reference group. **Results**: In a cohort of 824 patients, 25.1% were female (n = 207) and 74.9% were male (n = 617). The mean age was 64.1 years for females and 48.9 years for males. Those admitted for severe TBI were included in our analysis. Most patients were discharged home (52.8%), followed by death (12.4%), inpatient rehab (5.1%), and home with services (5.6%). Significant associations were found between disposition and sex, with both males and females most likely to be discharged home (*p* = 0.0174), as well as between disposition and injury type (*p* = 0.0186). Disposition was significantly associated with most major clinical variables: hospital length of stay (HLOS), vent days, Glasgow Coma Scale (GCS), and Injury Severity Score (ISS), with *p*-values < 0.0001 for ANOVA and non-parametric tests. Longer HLOS and ICULOS were associated with discharge to skilled nursing facilities (SNF) most frequently. Days on mechanical ventilation correlated most strongly with discharge to SNF. Lower GCS scores and higher AIS and ISS scores were linked to death or brain death. Prolonged EDLOS was predominantly associated with psychiatric admissions. Higher levels of ETOH were associated with discharge to police custody, followed by homelessness. **Conclusions**: Our study supports existing evidence that discharge disposition following severe TBI is influenced by several factors, such as injury severity, age, sex, and clinical variables, such as length of stay and ventilator days.

## 1. Introduction

### 1.1. Definition of Traumatic Brain Injury

The National Institute of Neurological Disorders and Stroke defines traumatic brain injury (TBI) as a brain injury that is caused by an outside force, such as a bump, blow, or jolt to the head or body [1]. Broadly, TBIs can be defined as penetrating, in which an object pierces the skull and enters the brain tissue, or non-penetrating, in which an external force is strong enough to move the brain within the skull. Further, TBI-related injury can be focal or diffuse and can range from mild concussions to more severe hematomas, contusions, skull fractures, or diffuse axonal injury (DAI) [1]. TBIs can additionally be classified as mild or moderate to severe, depending on a number of factors. Clinically, a mild TBI, or concussion, involves loss of consciousness lasting < 30 min, any alteration of consciousness, or post-traumatic amnesia lasting < 24 h. A Glasgow Coma Scale (GCS) score of 13 to 15 is also frequently used to classify mild TBI in a trauma setting, such as the ICU. In contrast, a moderate to severe TBI requires loss of consciousness lasting ≥ 30 min, post-traumatic amnesia lasting ≥ 24 h, or a GCS as low as 3 (a score of 9–12 for moderate TBI and < 8 for severe TBI) [2]. The severity of TBI can also be assessed by the presence of focal neurological signs and neuroimaging with CT or MRI [2].

### 1.2. Impact of TBI

#### 1.2.1. Demographics

Falls are the leading cause of TBI, especially among adults aged 65 and older, who are at the highest risk for hospitalization and death due to TBI. Males are more frequently hospitalized and have a threefold higher mortality risk compared to females [1]. A 2019 global study reported an 8.4% increase in age-standardized TBI prevalence and a 3.6% increase in incidence between 1990 and 2016, trends attributed to population growth, aging, and increased motor vehicle use [3]. Emergency departments in the U.S. handle over 25 million injury-related visits annually, including suspected TBIs [4]. In 2019, approximately 223,135 TBI-related hospitalizations and 64,362 TBI-related deaths were reported, with adults aged 75+ accounting for nearly one-third of both hospitalizations and deaths [4].

#### 1.2.2. Cost of TBI in the United States

Miller et al. (2021) estimated the annual incremental healthcare costs of nonfatal TBI in the U.S. at $40.6 billion in 2016, based on insurance claims data [5]. Although moderate to severe TBIs are more costly per individual, low-severity TBIs contribute to a higher total economic burden due to their greater frequency. The CDC estimates the lifetime economic cost of TBI in 2010 at $76.5 billion. These costs extend beyond acute care, as many patients experience chronic symptoms necessitating ongoing treatment [4].

#### 1.2.3. Factors Affecting Outcomes Following TBI

Moderate to severe TBI often results in lasting impairments affecting cognition, sensory processing, motor skills, mood, and behavior [6]. Five years post-injury, 57% of survivors remain moderately to severely disabled; 50% have at least one hospital readmission; 33% require assistance with daily activities; and 12% reside in nursing homes or institutions [6]. Longitudinal studies show variable recovery trajectories. Corrigan and Hammond (2013) found an increasing decline in global outcome categories over 15 years post-injury [7]. Forslund et al. (2019) observed that younger age, male sex, white-collar employment, and lower injury severity were associated with better global functioning over 10 years [8]. While the effects of sex on long-term outcomes are heterogeneous, recent analyses suggest females with moderate to severe TBI have higher in-hospital mortality, though no consistent sex differences are seen in long-term functional outcomes [9,10]. Disability, mental health, and quality of life outcomes are variably influenced by sex, age, and injury severity, complicating assessment of gender effects [9]. Domensino et al. (2024) reported that 3.2 years post-TBI, cognitive impairment, fatigue, and functional restrictions affect the majority of patients admitted to the ICU [11]. Humphries et al. (2022) identified low GCS, comorbidities, depression, and male sex as risk factors for poor outcome one year after mild TBI [12].

#### 1.2.4. Mechanism and Type of Injury

Mechanism of injury also impacts outcomes. In pediatric patients, abusive head trauma is linked to more severe injury and poorer prognosis than accidental injury [13]. In adults, age, GCS, and injury severity are stronger predictors of outcome than injury type [14,15]. Polytrauma further predicts worse discharge outcomes [15]. Overall, GCS, AIS, and ISS remain key prognostic indicators across populations [8,13].

#### 1.2.5. Alcohol Use and TBI

Chronic alcohol use before TBI is associated with poorer cognitive and neuropsychological recovery [16,17]. Roy et al. (2022) found that patients with alcohol use disorder had lower performance in language, memory, and executive functions following mild to moderate TBI [16]. A meta-analysis by Unsworth and Mathias (2017) reported poorer neuroimaging outcomes in TBI patients with a history of substance abuse but only moderate deficits in cognition [17]. In contrast, the impact of acute alcohol intoxication at injury time on outcomes is less clear. Mathias and Osborn (2018) found no consistent differences in long-term outcomes related to blood alcohol levels on admission [18]. Roy et al. (2022) similarly reported no acute cognitive recovery difference by blood alcohol level [16]. More recently, Jung et al. (2023) observed that pre-injury alcohol intake was associated with lower in-hospital mortality and better functional recovery in TBI patients, with higher alcohol concentrations correlating with lower mortality [19].

#### 1.2.6. Disposition Following TBI

Disposition after severe TBI strongly correlates with clinical outcomes. Discharge home is generally linked to better functional recovery and lower mortality, while discharge to long-term care is associated with older age, greater injury severity, and comorbidity burden [20,21,22]. Social determinants also influence disposition: white, non-Hispanic patients and those with insurance are more likely to be discharged to rehabilitation facilities [21,22,23]. Stanley et al. (2022) reported that hospital characteristics and regional practices affect disposition patterns, with larger hospitals and groups of neurosurgeons favoring skilled nursing discharges, and geographic variation influencing rates of home versus rehabilitation discharge [24].

Key gaps in the current literature include studies with large amounts of missing data and loss to follow-up. Many studies also often include smaller cohorts of patients, reducing their external validity. Older adults are often underrepresented, despite the high incidence of TBI in this group and the severity of outcomes. Additionally, current disposition decisions are often based on limited variables, such as CT findings, with insufficient integration of other potentially relevant factors.

This study aims to further correlate clinical outcomes with discharge dispositions in patients with severe TBI. Our cohort consisted of a large number of diverse patients at a level 1 trauma center, including a large fraction of adult and geriatric patients. Additionally, we analyze many clinical variables as they relate to disposition outcomes in detail, painting a much more comprehensive picture of the factors that influence patient disposition following TBI, such as the length of hospital stay, number of days on mechanical ventilation, and mean blood alcohol levels at admission. By addressing the gaps in the current literature, we hope to improve the post-TBI disposition process and make it more efficient and equitable.

## 2. Methods

We performed a single-center, retrospective review at Elmhurst Hospital, a Level 1 trauma center in Queens, New York City. The study included all patients who presented with severe TBI, defined as an AIS score of 3 or greater, between 1 January 2020 and 31 December 2023. Patients were excluded if they had non-severe or minor injuries, an AIS score less than 3, or died or were discharged within 24 h of admission.

Patient data were obtained from the National Trauma Registry of the American College of Surgeons (NTRACS) database at our institution. Patients were identified based on injury type (blunt vs. penetrating) and AIS score. After data review, a final cohort of 824 patients was included. Data extracted and organized in Excel encompassed demographics (sex, ethnicity, race), injury severity (GCS, AIS, ISS), injury type, mortality, disposition (e.g., home, rehabilitation, skilled nursing facility), length of stay (ED, hospital, ICU), ventilator days, and blood alcohol levels on admission.

All analyses were done using web-based SAS Studio OnDemand for Academics version 3.8 (v3.8). Bivariate associations between disposition and clinical variables were first assessed using ANOVA and non-parametric tests. Variables significant in univariate analysis were entered into a multinomial logistic regression model, with discharge home as the reference group. Covariates included age, gender, GCS, AIS (head), ISS, EDLOS, HLOS, ICULOS, ETOH, and mechanical ventilation. Adjusted odds ratios (ORs) and 95% CIs were reported. Model significance was assessed via Wald chi-square and likelihood ratio tests.

We used three methods of measuring injury severity in our analysis: the Glasgow Coma Scale (GCS), the Abbreviated Injury Scale (AIS), and the Injury Severity Score (ISS). The GCS is widely used in acute care and intensive care settings to objectively assess the level of consciousness in trauma patients. It evaluates three domains: eye-opening (scores 1–4), verbal response (scores 1–5), and motor response (scores 1–6), resulting in a total score ranging from 3 to 15 [25]. Since its introduction by Teasdale and Jennett in 1974, the GCS has become a standard measure of head injury severity. While its validity is generally supported by correlations with clinical and functional outcomes, reliability can vary based on assessor training and consistency. Among the components, motor response tends to have the highest interobserver reliability. Limitations include confounding factors, such as pre-existing neurological deficits, speech or hearing impairments, sedation, and intubation, which can restrict accurate assessment, highlighting the importance of early GCS evaluation [25,26].

The Abbreviated Injury Scale (AIS), developed in 1971 by the American Medical Association’s Committee on Medical Aspects of Automotive Injury, is an anatomical scoring system that rates injury severity in six body regions (head/neck, face, thorax, abdomen, extremities, and external) on a scale from 1 (minor) to 6 (maximal/fatal) using clinical and imaging findings [27]. Mild TBI corresponds to AIS head scores of 1–2, moderate TBI to 3–5, and fatal injuries to 6. Savitsky et al. (2016) identified an AIS head score of ≥5 as an optimal threshold for severe TBI [28]. Walder and Turnbull (1995) examined the correlation between AIS intracranial severity score and outcome following severe head injury by correlating the AIS head scores derived from CT scans of severe head injury patients with Glasgow Outcome Scale (GOS) [29]. They found that the AIS for head injury, based on initial CT scan, provides useful prognostic information in patients with severe head injury [29].

The Injury Severity Score (ISS), developed in 1974, is a widely used composite score derived from AIS ratings by summing the squares of the three highest AIS scores from different body regions [29,30]. An ISS > 15 is commonly used to define major trauma, though cutoffs may vary slightly depending on AIS versions [30].

## 3. Results

The study cohort included 824 patients, of whom 207 (25.1%) were female and 617 (74.9%) were male. The mean age was 64.1 years for females and 48.9 years for males (Table 1). Missing data ranged from approximately 24% to 27%, varying by variable. The majority of patients were discharged home (52.8%), followed by death (12.4%), discharge to home with services or subacute rehabilitation (SAR) (5.6%), and TBI rehabilitation (5.2%) (Table 1).

We found statistically significant associations between patient disposition and age in predicting “higher needs” dispositions, such as death, SAR, and TBI rehab (Table 2; died: OR = 1.105, 95% CI: 1.078–1.132, *p* < 0.0001; SAR: OR = 1.094, CI: 1.062–1.126, *p* < 0.0001; TBI Rehab: OR = 1.049, 1.025–1.074, *p* < 0.0001). Sex was found to be non-significant for most outcomes except “died” (Table 1; OR = 0.341, CI: 0.136–0.853, *p* = 0.0214). Females had lower odds of dying than being sent home compared to males. No significant association was observed between disposition and ethnicity (Table 1).

Disposition was significantly associated with GCS, ISS, HLOS, and Vent (Table 3). However, mean values of key clinical variables varied widely by disposition group. For example, patients discharged to SNF or TBI rehab had much longer hospital and ICU stays than those discharged home, while patients who died or were brain dead had higher ISS and AIS scores and lower GCS scores. Lower GCS scores at admission increased the odds of death (OR = 0.719, CI: 0.645–0.802, *p* < 0.0001). Higher ISS scores predicted higher odds of death (OR = 1.178, CI: 1.104–1.256, *p* < 0.0001), discharge to SAR (OR = 1.168, CI: 1.073–1.271, *p* = 0.0003), and discharge to TBI Rehab (OR = 1.114, CI: 1.041–1.193, *p* = 0.0018). HLOS was a significant predictor of discharge to SAR and SNF. The use of mechanical ventilation strongly increased the odds of “Brain Dead” (OR = 1.277), “Other Acute Care,” and “SNF” discharges. EDLOS, ICULOS, and EtOH were generally not significant predictors of disposition.

Table 4 illustrates the significant parametric (ANOVA) and non-parametric (Kruskal–Wallis) results for disposition compared to the length of stay data, with *p*-values < 0.0001 for each.

**Figure 1:**
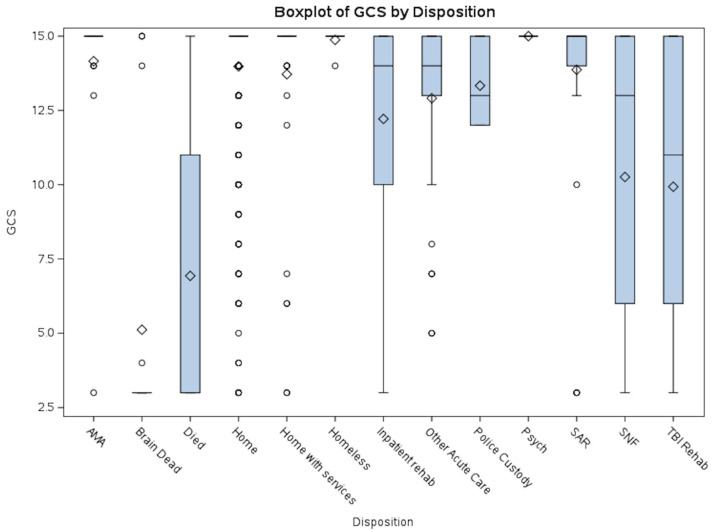
GCS vs. Disposition. This boxplot illustrates the distribution of average Glasgow Coma Scale (GCS) scores by disposition category. Lower GCS scores were predominantly observed in patients who died or were declared brain dead, whereas higher scores were associated with dispositions such as homelessness or discharge against medical advice (AMA). Patients discharged to Skilled Nursing Facilities (SNF), TBI Rehabilitation, or Inpatient Rehabilitation exhibited a broad range of GCS scores. Abbreviations: AMA = Discharged Against Medical Advice; SAR = Subacute Rehabilitation; SNF = Skilled Nursing Facility.

**Figure 2:**
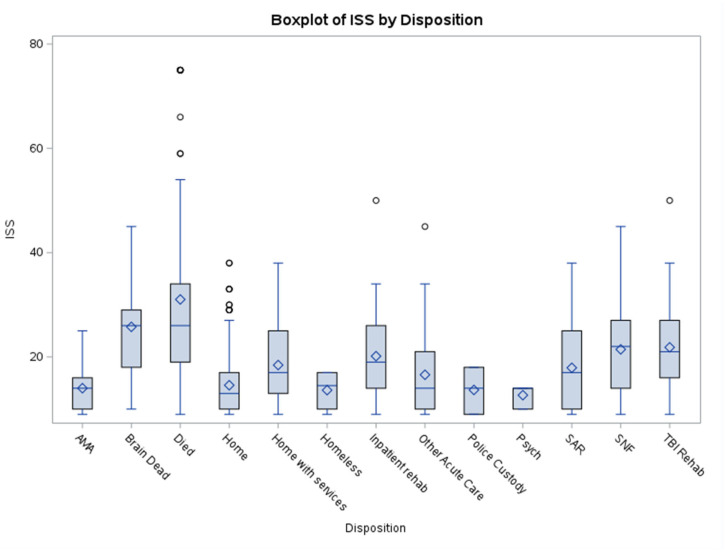
ISS vs. Disposition. This boxplot illustrates the distribution of average Injury Severity Score (ISS) by disposition category. Higher ISS scores were observed in patients who died, were declared brain dead, or were discharged to a skilled nursing facility (SNF). Lower scores were associated with discharge to psychiatric care, homeless, or police custody dispositions. Abbreviations: AMA = Discharged Against Medical Advice; SAR = Subacute Rehabilitation; SNF = Skilled Nursing Facility.

The boxplots of GCS vs. Disposition (Figure 1) and ISS vs. Disposition (Figure 2) help highlight case complexity and potential discharge bottlenecks by disposition. Discharge disposition strongly correlates with injury severity. Median ISS increases progressively from patients discharged home to those sent to rehab/SNF/SAR and is highest among those who died or were brain dead. Lower GCS and shorter EDLOS among brain-dead patients reflect streamlined clinical pathways. This information can inform process improvement efforts, especially targeting patients with prolonged EDLOS but stable neurologic function.

Overall, age, GCS, ISS, and HLOS emerged as significant independent predictors of discharge disposition (Table 5; global *p* < 0.01). Sex, AIS, ETOH, and EDLOS were not independently associated with disposition after adjustment. A one-point increase in ISS increased the odds of death by 17.8% (OR = 1.18; 95% CI: 1.10–1.26; *p* < 0.0001), discharge to SAR by 16.8% (OR = 1.17; 95% CI: 1.07–1.27; *p* = 0.0003), and discharge to TBI rehab by 11.4% (OR = 1.11; 95% CI: 1.04–1.19; *p* = 0.0018), compared to home discharge.

## 4. Discussion

Disposition has been extensively studied regarding TBI outcomes, with factors such as injury severity, mechanism of injury, age, and alcohol use playing critical roles in determining clinical outcomes [20,21,22,23,24]. In our study, outcomes were largely consistent with the existing literature. Both male and female patients were most likely to be discharged home. Longer hospital and ICU LOS were associated with discharge to inpatient facilities, such as SNF or TBI rehabilitation. However, while longer ED LOS is generally associated with increased likelihood of discharge to long-term care [21,22], we found increased ED LOS to be associated with discharge to psychiatric care, home with services, or police custody. Similarly, higher alcohol levels were most strongly associated with discharge to police custody, followed by homelessness, and then home. Patients on extended mechanical ventilation who survived without brain death were more likely to be discharged to inpatient facilities, such as SNF, TBI rehab, and inpatient rehab. Finally, the majority of our cohort sustained blunt injuries, which is the predominant cause of civilian TBI. Overall, age, GCS, ISS, and HLOS emerged as significant independent predictors of discharge disposition.

In our cohort, the most common disposition was discharge home, consistent with the existing literature [22,23,24,31,32], although a number of studies have noted that discharge home decreases with increased age [33,34]. We also observed a significant association between sex and disposition. Excluding patients who died, most female patients were discharged home, followed by discharge to subacute rehabilitation (SAR), and then home with services. In contrast, most male patients were discharged home, followed by TBI rehabilitation, and then inpatient rehabilitation or home with services in equal proportion. The relationship between sex and disposition post-TBI is complex and appears to interact with factors such as age, injury type and severity, alcohol use, and hospital characteristics.

Ingram et al. (2022) examined sex differences in trauma care efficiency and their association with discharge disposition using data from the 2013–2016 American College of Surgeons Trauma Quality Improvement Project (TQIP) database [35]. They reported that female patients experienced significantly longer emergency department (ED) length of stay (LOS) and delays in femur or pelvic fracture repairs and were more likely to be discharged to long-term care facilities than home after adjusting for age, injury severity score (ISS), injury type, and mechanism [35]. Similarly, in our analysis, although both males and females were most frequently discharged home, a significantly higher proportion of males (41%) were discharged home compared to females (12%), suggesting that female patients may be more likely to be discharged to long-term care facilities.

Teterina et al. (2023) investigated the impact of gender and sex on outcomes following severe TBI in a large cohort of Ontario residents [36]. They found that sex significantly influenced early mortality, while gender had a stronger effect on discharge disposition, with individuals expressing more “woman-like” characteristics having lower odds of discharge to rehabilitation versus home [36]. The observed differences in disposition may reflect social factors, including women’s traditional caregiving roles, reduced in-home support, or family preferences favoring institutional care for female patients [16,35,36]. Additionally, female injury severity may be underestimated, leading to under-triage and less aggressive acute care, which could influence disposition away from home [34]. Such sex-based differences have been reported across a range of acute and chronic conditions, including general trauma and hypoxic-ischemic brain injury [35,37].

Length of stay in the hospital, ED, and intensive care unit (ICU) were all significantly associated with disposition in our study. Consistent with prior research, longer hospital LOS is strongly linked to discharge to skilled nursing facilities, long-term care, or inpatient rehabilitation, whereas shorter LOS correlates with discharge home; these relationships persist after controlling for age, injury severity, comorbidities, injury mechanism, and hospital factors [21,22]. Taylor et al. (2024) defined prolonged LOS as greater than two standard deviations above the mean (24 days) following TBI and found that such patients were more likely to be discharged to inpatient facilities or to die and less likely to be discharged home [38]. These patients also presented with lower Glasgow Coma Scale (GCS) scores on arrival, longer ICU stays, higher ISS, and increased ventilator days [38]. Our findings align with these observations: longer hospital and ICU LOS were associated with discharge to inpatient facilities, such as SNF or TBI rehabilitation, while extended ED LOS correlated with discharge to psychiatric care, home with services, or police custody.

Yue et al. (2024) identified Medicaid insurance as an independent predictor of prolonged hospital LOS (>47 days) across TBI severity strata [39]. The effect size of Medicaid on prolonged LOS increased with injury severity, and paradoxically, patients with more severe injuries who would benefit from post-acute care faced greater risks of delayed discharge [39]. Although we did not analyze the insurance status in our cohort, its potential modifying effect on disposition warrants further investigation.

Consistent with the existing literature, longer ICU LOS was associated with reduced likelihood of discharge home and increased likelihood of discharge to skilled nursing facilities, long-term care, or rehabilitation [21,22,23,40,41]. Beijer et al. (2023) reported that female patients with severe TBI admitted to level 1 trauma centers in the Netherlands had lower ICU admission rates and shorter ICU LOS, with female sex associated with a decreased likelihood of ICU stays exceeding seven days [42].

While increased ED LOS is generally associated with decreased likelihood of discharge home and increased likelihood of discharge to long-term care [20,21,22,23,24], our study’s findings differed somewhat. The longest average ED LOSs were observed among patients discharged to psychiatric care or police custody, likely reflecting a high prevalence of undomiciled patients and elevated blood alcohol levels at admission. Elevated alcohol levels were most strongly associated with discharge to police custody, followed by homelessness, and then home. Prior studies similarly report that higher blood alcohol levels at presentation correlate with decreased likelihood of discharge home. These patients tend to be younger, male, and present with lower GCS and higher ISS, increasing their likelihood of institutional care at discharge [23,43]. Blood alcohol level is generally a marker for more severe injury and higher probability of discharge to long-term care, with these associations modulated by demographic and clinical factors [19,43,44].

Patients who required prolonged mechanical ventilation and survived without brain death were more likely to be discharged to SNF, followed by TBI rehabilitation and inpatient rehabilitation. This aligns with existing data demonstrating that prolonged ventilation is a marker of increased injury severity and clinical complexity, reducing the likelihood of functional independence at discharge. Prolonged mechanical ventilation is also associated with extended ICU and total hospital LOS in severe TBI patients [45,46], making it a robust indirect predictor of disposition.

Finally, the type of injury was significantly associated with disposition. Approximately 98% of patients sustained blunt injuries, with the majority discharged home, followed by death, SAR, and home with services equally. Among the 18 patients with penetrating injuries, nearly all were discharged home or died (six each), although the small sample limits definitive conclusions. While blunt trauma predominates as a cause of civilian TBI [47], current evidence does not support injury type as an independent predictor of disposition or outcomes.

While injury severity indices, such as Glasgow Coma Scale (GCS), Abbreviated Injury Scale (AIS), and Injury Severity Score (ISS), as well as hospital length of stay (LOS), remain robust independent predictors of post-TBI disposition in the available literature, factors such as sex are influenced by a combination of clinical and social factors, including age, pre-existing comorbidities, and support systems, which collectively affect outcomes and discharge planning. Our findings emphasize the importance of considering multiple patient and clinical characteristics when predicting discharge disposition.

### Strengths and Limitations

A key strength of this study is the comprehensive analysis of a large, diverse patient cohort with severe TBI, allowing examination of multiple factors—including injury severity, sex, length of stay across different hospital settings, and blood alcohol levels—and their associations with discharge disposition. Additionally, the integration of detailed clinical variables alongside disposition outcomes provides valuable insights consistent with, and complementary to, the existing literature.

However, this study has several limitations. The retrospective design limits causal inference and is subject to potential biases inherent in registry and medical record data. Insurance status was not evaluated, despite evidence from prior research suggesting that insured, white, and non-Hispanic patients are more frequently discharged to long-term rehabilitation facilities rather than home, and may have confounded observed associations. Notably, we did not observe statistically significant differences in disposition based on ethnicity, which may reflect limitations in sample size or demographic composition.

The small sample size of penetrating injury cases restricts meaningful comparisons with blunt injury. Furthermore, social determinants, such as caregiver availability, socioeconomic status, and pre-existing comorbidities—factors known to affect discharge disposition—were not fully captured in the dataset. Additionally, potential variability in hospital discharge practices and resource availability at a single center may limit the generalizability of our results to other settings.

Future prospective multicenter studies incorporating insurance data, detailed social factors, and broader clinical variables would provide a more comprehensive understanding of the complex interplay of factors influencing disposition outcomes following severe TBI. This would also help clarify the nuanced role of sex and other demographic factors observed in this study.

## 5. Conclusions

Our analysis corroborates existing literature demonstrating that discharge disposition following severe traumatic brain injury (TBI) is multifactorial. We concluded that older age, higher ISS, lower initial GCS, and longer hospital stays significantly increase the odds of adverse or higher-care dispositions (e.g., death, SAR, SNF, rehab) over being sent home. Sex only impacts the odds of death. Other variables like alcohol (EtOH) and length of time in various units (EDLOS, ICULOS) were not strong predictors in this sample. Future research should prioritize comprehensive, prospective studies that include socioeconomic and psychosocial determinants, enabling the development of predictive models with greater accuracy and clinical utility. Such efforts may ultimately inform individualized discharge planning and optimize resource allocation for this vulnerable population.

## Figures and Tables

**Table 1:** Demographics.

	AMA	Brain Dead	Died	Home	Home with Services	Homeless	Inpatient Rehab	Other Acute Care	Police Custody	Psych	SAR	SNF	TBI Rehab	Total
**Sex (n,%)**														
Female	2 (0.24)	2 (0.24)	26 (3.16)	99 (12.01)	18 (2.18)	-	14 (1.70)	7 (0.85)	-	2 (0.24)	19	7 (0.85)	11 (1.33)	207 (25.12)
Male	17 (2.06)	15 (1.82)	76 (9.22)	336 (40.78)	28 (3.40)	8 (0.97)	28 (3.40)	26 (3.16)	3 (0.36)	1 (0.12)	27 (3.28)	20 (2.43)	32 (3.88)	617 (74.88)
**Ethnicity**														
Hispanic Origin	8	10	41	214	19	4	16	14	2	1	16	9	17	371
Non-Hispanic Origin	11	6	51	200	22	4	25	18	1	2	29	16	23	408
Unknown	-	1	10	21	5	-	1	1	-	-	1	2	3	45
**Race**														
Asian	2	1	19	58	8	-	6	7	-	2	8	3	7	121
Black	4	1	3	37	6	4	4	3	1	.	3	2	1	69
Native Hawaiian or Other Pacific Islander	-	-	1	1	-	-	1	-	-	-	-	-	-	3
Other	9	10	51	269	19	4	23	17	2	1	25	15	23	468
Unknown	-	1	8	8	1	-	-	1	-	-	-	-	2	21
White	4	4	20	62	12	-	8	5	-	-	10	7	10	142
**Type of Injury**														
Blunt	19	15	96	429	46	8	42	33	3	3	46	25	41	806
Penetrating	-	2	6	6	-	-	-	-	-	-	-	2	2	18

Demographic characteristics of the 824 patients included in the analysis. The cohort was predominantly male (n = 617), non-Hispanic (n = 408), identified as “Other” race (n = 468), and sustained blunt injuries (n = 806). Abbreviations: AMA = Discharged Against Medical Advice; SAR = Subacute Rehabilitation; SNF = Skilled Nursing Facility. Bold is to show the main headings in this table.

**Table 2:** Age Group vs. Disposition.

	AMA	Brain Dead	Died	Home	Home w/Services	Homeless	Inpt Rehab	Other Acute	Police	Psych	SAR	SNF	TBI Rehab	Total
**Infant (0 ≤ years < 2)**	.	.	.	2	.	.	.	7	.	.	.	.	.	9
**Child (2 ≤ years < 13)**	.	.	2	2	.	.	.	3	.	.	.	.	.	7
**Adolescent (13 ≤ years < 18)**	.	.	.	5	.	.	1	1	.	.	.	.	.	7
**Adult (18 ≤ years < 65)**	15	13	56	328	17	7	23	12	3	3	10	17	33	537
**Geriatric (≥65 years)**	4	4	44	98	29	1	18	10	.	.	36	10	10	264

This is a descriptive table categorizing dispositions by age group (infant, child, adolescent, adult, child). Most patients were either in the adult (n = 537) or geriatric (n = 264) categories. Abbreviations: AMA = Discharged Against Medical Advice; SAR = Subacute Rehabilitation; SNF = Skilled Nursing Facility. Bold is to show the main headings in this table

**Table 3:** Mean clinical variables vs. Disposition.

Disposition	N	Age (Mean Years)	EDLOS (Mean Hours)	HLOS (Mean Days)	ICULOS (Mean Days)	Vent (Mean Days)	GCS (Mean Score)	ISS (Mean Score)	AIS (Mean Score)	ETOH (Mean mg/dL)
**AMA**	19	55.15	13.89	6.63	3.28	1.26	14.16	14	3.32	104.47
**Brain Dead**	17	53.18	5.29	11.24	4.49	8.65	5.12	25.76	4.47	98.18
**Died**	102	58.99	4.88	9.05	4.35	2.77	6.93	31.02	4.24	79.24
**Home**	435	48.13	14.5	6.6	1.77	0.41	13.98	14.57	3.41	114.79
**Home with services**	46	65.13	16.44	12.76	2.89	0.59	13.72	18.43	3.87	40.75
**Homeless**	8	49.65	12.64	7.63	0.26	0	14.88	13.63	3.38	162.38
**Inpatient rehab**	42	59.59	10.23	18.33	7.67	2.71	12.21	20.14	3.79	66.22
**Other Acute Care**	33	39.43	9.24	8.64	3.15	1.91	12.91	16.58	3.67	45.43
**Police Custody**	3	30.93	15.79	3.67	2.44	0	13.33	13.67	3	288.67
**Psych**	3	34.13	19.52	8.33	1.74	0	15	12.67	3	0
**SAR**	46	75.08	12.5	22.65	3.51	0.76	13.87	17.91	3.65	39.72
**SNF**	27	54.06	9.19	50.89	15.58	11.11	10.26	21.44	4	89.2
**TBI Rehab**	43	52.22	7.6	35.6	11.53	5.23	9.93	21.84	3.98	108.24

This table presents the total number of patients within each disposition category, alongside mean values for key clinical variables stratified by disposition. Patients with longer hospital length of stay (HLOS) and intensive care unit length of stay (ICULOS) were more likely to be discharged to skilled nursing facilities (SNF), followed by traumatic brain injury (TBI) rehabilitation. The extended duration of mechanical ventilation correlated most strongly with discharge to SNF and brain death. Lower Glasgow Coma Scale (GCS) scores and higher Abbreviated Injury Scale (AIS) and Injury Severity Score (ISS) values were linked to death or brain death. Elevated mean blood alcohol levels were more likely to result in discharge to police custody, followed by homelessness. Prolonged emergency department length of stay (EDLOS) was predominantly associated with psychiatric admissions and police custody. Abbreviations: EDLOS = Emergency Department Length of Stay; HLOS = Hospital Length of Stay; ICULOS = Intensive Care Unit Length of Stay; GCS = Glasgow Coma Scale; ISS = Injury Severity Score; AIS = Abbreviated Injury Scale; ETOH = Blood ethanol level on admission; AMA = Discharged Against Medical Advice; SAR = Subacute Rehabilitation; SNF = Skilled Nursing Facility. All lengths of stay and ventilation duration are reported in days; ETOH is reported in mg/dL. Bold is to show the main headings in this table.

**Table 4:** ANOVA/Kruskal–Wallis results for key variables.

Variable	Test	F/Chi-Square	DF	*p*-Value	Significant?
**EDLOS**	ANOVA	8.14	12	<0.0001	Yes
**HLOS**	ANOVA	17.37	12	<0.0001	Yes
**ICULOS**	ANOVA	17.03	12	<0.0001	Yes
**Vent**	ANOVA	14.03	12	<0.0001	Yes
**GCS**	ANOVA	43.77	12	<0.0001	Yes
**ISS**	ANOVA	27.25	12	<0.0001	Yes
**AIS**	ANOVA	13.48	12	<0.0001	Yes
**ETOH**	ANOVA	3.08	12	0.0003	Yes

This table presents the results of parametric (ANOVA) and non-parametric (F test/chi-Square) analyses comparing key clinical variables across disposition groups. All variables demonstrated statistically significant differences with *p*-values < 0.001. Abbreviations: EDLOS = Emergency Department Length of Stay; HLOS = Hospital Length of Stay; ICULOS = Intensive Care Unit Length of Stay; GCS = Glasgow Coma Scale; ISS = Injury Severity Score; AIS = Abbreviated Injury Scale; ETOH = Blood ethanol level on admission; DF = Degrees of Freedom. Bold is to show the main headings in this table.

**Table 5:** Global tests.

Predictor	Degree of Freedom (DF)	Wald Chi-Square	*p*-Value	Interpretation
**Age**	12	98.2	<0.0001	Strong overall effect
**Sex**	12	12.2	0.432	Not significant
**GCS**	12	50.7	<0.0001	Strong overall effect
**AIS**	12	9.6	0.650	Not significant
**ISS**	12	32.6	0.0011	Significant effect
**HLOS**	12	40.2	<0.0001	Strong effect
**EDLOS, ICULOS, ETOH, Vent**	Not significant overall			

Age, GCS, ISS, and HLOS are highly significant predictors of discharge disposition. Sex, AIS, EDLOS, ICULOS, ETOH, and Vent days are not significant overall. Abbreviations: GCS = Glasgow Coma Scale; AIS = Abbreviated Injury Scale; ISS = Injury Severity Score; HLOS = Hospital Length of Stay; EDLOS = Emergency Department Length of Stay; ICULOS = Intensive Care Unit Length of Stay; ETOH = Blood Ethanol Level on Admission. Bold is to show the main headings in this table.

## Data Availability

The data was requested from the Elmhurst Trauma registry and extracted using electronic medical records after receiving approval from the Institutional Review Board at our facility (Elmhurst Hospital Center).
